# Catastrophic *Candida* prosthetic valve endocarditis and COVID-19 comorbidity: A rare case

**DOI:** 10.18502/cmm.7.2.7157

**Published:** 2021-06

**Authors:** Lotfollah Davoodi, Leila Faeli, Rogheye Mirzakhani, Rozita Jalalian, Tahereh Shokohi, Firoozeh Kermani

**Affiliations:** 1 Antimicrobial Resistance Research Center, Communicable Diseases Institute, and Department of Infectious Diseases, Mazandaran University of Medical Sciences, Sari, Iran; 2 Student Research Committee, Mazandaran University of Medical Sciences, Sari, Iran; 3 Department of Cardiology, Mazandaran University of Medical Sciences, Sari, Iran; 4 Invasive Fungi Research Center, Communicable Diseases Institute, Mazandaran University of Medical Sciences, Sari, Iran; 5 Department of Medical Mycology, School of Medicine, Mazandaran University of Medical Sciences, Sari, Iran

**Keywords:** *Candida* endocarditis, Case report, Comorbidity, COVID-19, Prosthetic valve replacement, SARS-CoV-2, Transesophageal echocardiography

## Abstract

**Background and Purpose:**

Coronavirus disease 2019 (COVID-19) and *Candida* prostatic valve endocarditis present various clinical manifestations which may overlap; hence, discrimination between them is extremely difficult.

**Case report:**

The case was a 66-year-old man with a past medical history of mitral and aortic valves replacement one year before COVID-19 co-infection. He was admitted with fever (for 7 days), shortness of breath, cough, seizure, lethargy, headache, and 85% oxygen saturation. Transesophageal echocardiography revealed multiple large-sized, highly mobile masses on both sides of the mechanical mitral valve highly suggestive of vegetation. Chest computed tomography scanning showed simulating scattered COVID-19 peripheral ground-glass opacities confirmed by reverse-transcription polymerase chain reaction. The set of blood cultures yielded yeast colonies that were identified as *Candida tropicalis*. The patient died of septic shock shortly after receiving antifungal therapy.

**Conclusion:**

This case emphasized the importance of early diagnosis and implementation of antifungal treatment, particularly in patients with prosthetic cardiac valves, to reduce their unfavorable outcomes in COVID-19 patients

## Introduction

Coronavirus disease 2019 (COVID-19) is a novel coronavirus that caused respiratory tract infection of unknown sources on December 31, 2019, in Wuhan, Hubei, China. COVID-19 can range from asymptomatic to severe [ 1 ]. Its most common symptoms are fever (98%), cough (76%), dyspnea (55%), myalgia, and fatigue (44%) [ 1 , 2 ]. In some COVID-19 patients, gastrointestinal involvement, acute cardiac, and acute kidney injuries are also reported. Moreover, some neurological symptoms, including dizziness, headache, hypogeusia, and hyposmia are found as well. Severe COVID-19 cases are accompanied by both ischemic stroke and intracerebral hemorrhagic stroke and loss of consciousness [ 3 , 4 ]. 

Cardiovascular disease is one of the most frequent comorbidities in COVID-19 patients which requires hospitalization, has a higher case fatality rate, and is more severe [ 5 ]. Endocarditis is a severe infectious disease with a high mortality rate [ 2 - 5 ]. Its in-hospital mortality rates are 12-17%, 23%-26%, 10%, and 8% for inherent valve, prosthetic valve, cardiac implantable electronic device, and right-sided endocarditis, respectively [ 6 ]. Fungal endocarditis in patients with prostatic valves can be a severe threat with high mortality and morbidity [ 7 ]. The immune dysregulation associated with COVID-19 contributes to microbial proliferation and the development of infection.

There are few reports of COVID-19 cases with concomitant fungal endocarditis [ 7 , 8 ]. The clinical manifestations of infective endocarditis and COVID-19 overlap, including fever, dyspnea, fatigue, cough, and myalgia; hence, its differential diagnosis can be challenging. Therefore, the initial screening of COVID-19 concomitant with infective endocarditis is ambiguous. 

The present study presents a catastrophic case of fungal prosthetic valve endocarditis (PVE) and COVID-19 comorbidity. Every clinician, especially those in developing countries, should consider echocardiography when evaluating the COVID-19 patients with a prosthetic heart valve.

## Case report

**Figure 1:**
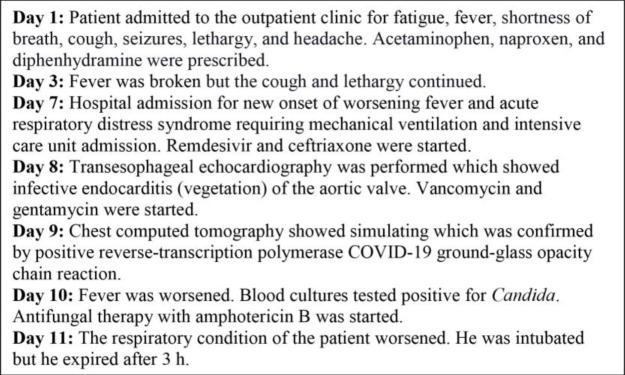
Timeline of the illness of the patient.

In July 2020, a 66-year-old man with a 7-day fever, shortness of breath, cough, seizure, lethargy, headache, and blood oxygen saturation (SpO_2_) of 85% was admitted to the Emergency Department of Fatemeh Zahra Hospital affiliated to Mazandaran University of Medical Sciences, northern Iran. The timeline of his illness is illustrated in Figure 1. Laboratory testing in the onset of symptom (before hospital admission) showed a C-reactive protein (CRP) level of 43 mg/L, white blood cell (WBC) count of 2.65×10^9^/L, neutrophil 76%, lymphocyte 22%, hemoglobin 11.3 g/dL, mean corpuscular volume of 79 fL, erythrocyte sedimentation rate (ESR) of 76 mm/h, red blood cell count of 3.95×10^6^/ μl, and lactate dehydrogenase 877 U/L.

Acetaminophen (2 g/day), naproxen (500 mg every 12 h), diphenhydramine compound (300 mg/day) were prescribed at the outpatient clinic. The fever stopped after three days; however, the cough and lethargy continued. His past medical history revealed aortic valve and mitral valve replacement (AVR, MVR) with mechanical prosthetic valve two years before his hospital admission. Moreover, it was found that he has been receiving warfarin (5 mg/day), losartan (25 mg/day), and furosemide (25 mg twice a day). 

On the first day of admission, laboratory examinations showed CRP 65 mg/L, WBC count of 1.43×10^9^/L, neutrophil 66%, lymphocyte 31%, ESR 76 mm/h, and the international normalized ratio of 2.3. On the first day of hospitalization, the patient received 0.9% sodium chloride injection in addition to intravenous acetaminophen and parenteral vitamin C (2 g/day). 

**Figure 2:**
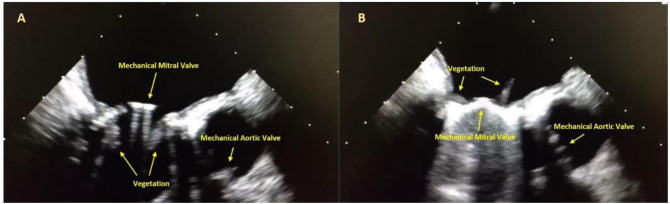
**A, B.** Transesophageal echocardiography examination in 120° showed mechanical prosthetic bileaflet mitral valve with multiple large size vegetation (arrow) on both sides of prosthetic valves.

On the second day, due to persistent fever, transesophageal echocardiography (TEE) examination was performed. Both mechanical prosthetic mitral and aortic valves had normal function and motion as well as an acceptable gradient. The TEE also revealed multiple large size highly mobile masses on both sides of the mechanical mitral valve protruding to the left atrial cavity that was highly suggestive of vegetation (figures 2A and B). Due to the presence of mechanical prosthetic valve vegetation, vancomycin (2 g/day) and gentamicin (6 mg/kg bw/day) were added to the treatment. 

On the third day of admission, spiral lung computed tomography scanning showed simulating scattered COVID-19 peripheral ground-glass opacities. The COVID-19 diagnosis was confirmed by the positive result of reverse-transcription polymerase chain reaction (RT-PCR) for COVID‐19; therefore, remdesivir (200 mg loading dose on day 1, followed by 100 mg daily mg), ceftriaxone (2 g/day), and fluticasone propionate (250 μg twice daily) were administered. Due to prolonged fever, a set of blood cultures were ordered as well.

On the third day, despite receiving oxygen by reservoir mask, his oxygen saturation (SpO_2_) was 89%. The growth in blood culture bottles was checked by sub-culturing onto sabouraud dextrose agar (Laboratorios Conda, Madrid, Spain), and after being kept at 30 ºC for 48 h it yielded yeast colonies. The smooth white-to-cream colonies showed hyaline budding yeast cells that were identified as *Candida* species. Liposomal amphotericin B was started immediately. 

On the night of the fourth day, the patient's respiratory condition worsened. He was intubated immediately; however, he was asystole after 3 h of intubation. Cardiopulmonary resuscitation quickly began, but the attempt was not successful. The routine workup was performed on yeast colonies to identify the causative agent. The obtained colonies were sub-cultured on CHROMagar *Candida* medium (Merck, Germany) and yielded blue colonies, presumably *Candida tropicalis*. 

For confirmation, PCR assay was performed using the two universal primers internal transcribed spacer 1 (ITS1) and ITS4 [ 7 ]. The ITS region (ITS1-5.8S, ITS2 rDNA) was amplified. The amplicon was sequenced (Gen Fanavaran Ltd., Tehran, Iran) and compared with sequences deposited in GenBank and The Dutch Centraalbureau voor Schimmelcultures (CBS), databases which showed 100% similarity with the *C. tropicalis*. The obtained sequence was deposited at the GenBank database under the accession number MW911803. 

The *in vitro* antifungal susceptibility of the obtained isolate was performed according to the Clinical and Laboratory Standards Institute documents M27-A4 and M27-S3. Minimum inhibitory concentration results showed the isolate was susceptible to fluconazole (1 μg/ml), voriconazole (0.063 μg/ml), posaconazole (0.5 μg/ml), amphotericin B (0.25 μg/ml) and itraconazole (0.5 μg/ml). However, the isolate was resistant to caspofungin (4 μg/ml), anidulafungin (2 μg/ml), and micafungin (4 μg/ml).

## Discussion

COVID-19 and PVE present various clinical manifestations that may overlap, including fever, shortness of breath, cough, seizure, lethargy, headache, and myalgia. Due to severity, manifestations of these diseases are misleading and can lead to grave consequences. To the best of our knowledge, there is no other report describing fatal *C. tropicalis* bloodstream infection in a patient with COVID-19 in Iran. According to the literature, the prevalence of fungal infections, like candidiasis, is high in COVID-19 patients [ 8 ]. *Candida tropicalis* is one of the most common *Candida* causing human disease in tropical countries. *Candida tropicalis* is the most common leading cause of candidemia in countries, such as Algeria, the United Arab Emirates, Qatar, India, and Iran, and has high mortality rates [ 8 - 11 ].

The recent systematic review in Iran has shown that *C. tropicalis* is the fourth causative agent of candidemia [ 11 ]. It is one of the most prevalent microbiotas of the healthy human gastrointestinal tract [ 12 ] and the primary cause of candidemia [ 13 ] with a high mortality rate and a higher rate of fluconazole resistance [ 14 ]. Many studies have been carried out on the virulence-associated markers of *C. tropicalis*, such as biofilm formation and secretion of proteinases [ 15 ], that help them be adherent to most tissues and create invasion and dissemination. Recently, Sasani et al. [ 16 ] in their study isolated 39 *C. tropicalis* strains from patients with candidemia. They found an association between *C. tropicalis* biofilm formation and the increasing mortality rates.

It is crucial to note the urgent need for screening candidemia in critically ill COVID-19 patients in a high-risk setting. The (1-3)-β-D-Glucan with high negative predictive value was presented by White et al. as a basic screening test [ 17 ] for detecting invasive *Candida* infection and other invasive fungal infections. However, the rapid in-house version is not available in most hospitals for qualitative detection in serum. 

Further evaluation by blood culture is limited by long turnaround time, expert personnel, and lower sensitivity. In such case, in-house PCR, the commercial panel, including the T2 *Candida* panel (T2C; T2 Biosystems, Lexington, MA, USA), allows rapid species-specific detection and efficient diagnosis of candidemia to direct targeted therapy although the technical demand may be a bug [ 18 ]. 

Combination of (1-3)-β-D-Glucan and Platelia *Candida*-specific antigen (Bio-Rad Laboratories, Marnes-la Coquette, France) tests to increase the sensitivity and specificity of the diagnosis of invasive candidiasis which can be increased even more when combined with procalcitonin for differentiating fungal from bacterial etiology [ 19 ]. Usage of the above-mentioned techniques remains determined in COVID-19 patients with acute respiratory distress syndrome [ 18 ].

The evidence from this study suggested that the COVID-19 pandemic has outlined new challenges for the diagnosis of cardiovascular diseases and subsequent care and treatment. *Candida* bloodstream infection, particularly those caused by resistant agents to the common antifungals, has been reported in patients with severe COVID-19 [ 20 ].

Some patients have suspected or confirmed SARS COV-2 infection with a history of prosthetic valve replacement and high clinical suspicion of infective endocarditis, including persistent fever and a high level of inflammatory markers. For these patients, it is crucial to perform echocardiography, preferably transesophageal with appropriate preventive precautions for identifying vegetation and intracardiac complications. Echocardiography contributes to the improvement of patient care and may provide life-saving interventions. 

Clinicians should consider the possibility of candidemia in patients with severe COVID-19 who present persisting fever and clinical deterioration. In the case presented in the present report, positive blood cultures during fever and TEE showing vegetation on prosthetic cardiac valve helped diagnose PVE. However, despite the antifungal treatments, the patient expired probably due to progressive *Candida* endocarditis.

## Conclusion

This report highlighted a fatal COVID-19 and Candida endocarditis comorbidity in a patient with prosthetic valve replacement as a rare clinical entity. This should attract the attention of physicians toward the possibility of endocarditis in COVID-19 patients with recent cardiac surgery who presented early with acute heart failure. Given the high mortality rate of this co-infection, the early diagnosis and timely antifungal therapy can improve the survival rate. Epidemiological studies performed to evaluate the occurrence and impact of these co-infections are highly recommended to increase physician awareness about the outcome of COVID-19 in this regard.

## Ethical considerations

The Ethics Committee of Mazandaran University of Medical Sciences, Sari, Iran, approved this report (IR.MAZUMS.REC.1399.551). Written informed consent was obtained from the legal guardian for the inclusion of details in the manuscript and publication.

## Acknowledgement

The author would like to thank the Invasive Fungi Research Center of Mazandaran University of Medical Sciences, Sari, Iran, for financial support (Grant No. 1327). 

## Authors’ contribution

T. S. and L. D. conceived, designed, and coordinated the research. L.F., R.M., and R.J. collected data. T. S., L. D., and L.F. wrote the paper. All authors revised the manuscript, contributed to the improvement of the paper, and also read and approved the final manuscript.

## Conflict of Interest

The authors declare no conflicts of interest regarding the publication of this paper.

## Financial disclosure

No financial interests related to the material of this manuscript have been declared.
